# High-Throughput Preparation and Machine Learning Screening of a Blue-Phase Liquid Crystal Based on Inkjet Printing

**DOI:** 10.3390/molecules27206938

**Published:** 2022-10-16

**Authors:** Wan-Li He, Yong-Feng Cui, Shi-Guang Luo, Wen-Tuo Hu, Kai-Nan Wang, Zhou Yang, Hui Cao, Dong Wang

**Affiliations:** Department of Materials Science and Engineering, University of Science and Technology Beijing, Beijing 100083, China

**Keywords:** blue-phase liquid crystal, high throughput, inkjet printing, machine learning, blue-phase temperature range

## Abstract

Blue-phase liquid crystal (BPLC) is considered as the next-generation liquid crystal display material, but its practical application is seriously affected by a narrow temperature range and a long research period. In this paper, we used inkjet printing technology to prepare BPLC materials with high throughput, and try to use machine vision technology to test BPLC with high throughput. The “standard curve method” for establishing each printing channel and the “vector matching method” for searching the chromaticity value of the minimum distance were proposed to improve the accuracy of inkjet printing BPLC materials. For a large number of sample-phase images, we propose a machine learning method to identify the liquid crystal phase. In this paper, for the first time, the high-throughput preparation and high-throughput detection of 1080 BPLC samples with five common components by a comprehensive experimental method has been successfully realized. The results are helpful to improve the research efficiency of blue-phase materials and provide a theoretical basis and practical guidance for rapid screening of multi-component BPLC materials.

## 1. Introduction

Blue-phase liquid crystal (BPLC) has the advantages of a wide viewing angle, no directional layer and a sub millisecond response speed when applied in the display field. It was once considered as a candidate material for the next generation of optical devices [1,2]. However, the temperature range of BPLC is too narrow or the driving voltage is too high to be used for actual display. Therefore, BPLC materials have been attracting the attention of extensive researchers. Since the blue phase usually exists in a narrow temperature range (0~5 K) below the LC clear point, researchers need to slow down the temperature change rate (generally 0.5 k/min) before entering the blue-phase range in order to accurately observe the phase transition behavior, so that the observation period of the blue phase is longer than that of other phase liquid crystals. Therefore, the detection efficiency of all the properties of the BPLC is very low. Compared with other LC materials, the preparation period of BPLC is also longer. The blue phase is also relatively sensitive to component content and external temperature conditions, so it is easy to encounter errors in the process of preparation, mixing and detection. Therefore, in order to improve the accuracy of preparation and detection, it is necessary to prepare or test the same sample repeatedly when necessary, which further extends the research time. Since the preparation process of the BPLC sample and the procedure for testing its performance are sequential iterations, when using the above traditional “step-by-step” mode of research method to prepare and test multiple blue-phase samples with different components, there are lengthy experimental steps, which is time-consuming and has low efficiency, making it difficult to meet the needs of rapid and large-scale sample preparation and testing. Therefore, developing a time-saving and efficient screening method has become a very urgent problem to be solved.

Since the Materials Genome Initiative (MGI) was proposed, high-throughput technologies have been widely used in the field of materials science [3,4] Fundamentally, high-throughput technology is a concurrent research method, including synthesis, preparation, detection and characterization technology, which often generates a large amount of data, enabling researchers to obtain more results from fewer experiments, Therefore, it can speed up the experimental progress and shorten the research time [5]. This paper attempts to apply high-throughput technology to BPLC materials to improve the research efficiency of BP materials, so as to break through the bottleneck of BPLC materials and promote its practical application.

How to prepare the BPLC Library in batch and quickly detect its temperature range and other properties are important problems to be solved in the process of applying high-throughput technology to BPLC materials. According to the characteristics of component distribution, high-throughput sample preparation technology is classified into sample library preparation and continuous component preparation. The former is usually used to prepare samples in high-throughput. It is mainly based on the content of combinatorial theory, and forms a discrete sample array library by mixing different amounts of precursor materials, components and controlling reaction conditions. In 1995, Xiang prepared a sample library containing 128 sample points on the substrate by combining physical deposition with binary mask [4]. Fujimoto et al. adjusted the conveying speed of the solution feed to control the components of the mixed solution [6], so as to realize the mixing of raw materials in different proportions, and finally obtained a series of samples with different components through atomization, deposition, drying and other steps.

In addition, although the robot arm is directly used to mix different proportions of materials [7], the cost of the robot arm is huge, which is difficult to achieve in a conventional laboratory. Fortunately, inkjet printing technology is a promising method for building material libraries, which is often used to prepare material libraries in the microfluidic field [8] and enzyme detection field [9]. The main inkjet printing technology for material mixing is piezoelectric inkjet printing (IJP) technology. Generally speaking, the control parameters of the printer need to be adjusted due to the different fluid properties of different materials before the material library construction [10]. In order to make each material eject according to the predicted quantity, each material needs to set specific parameters. This method has been used in liquid crystal mixing [11,12,13] and solar cell preparation [14], but the steps of parameter adjustment are very complicated. The other method needs to adjust the viscosity, surface tension and other fluid characteristics of the ink to match the ink produced by the original printer manufacturer [15,16,17], but these processes are also complicated and take a long time. Therefore, if there was a cheaper and more available way to print materials, which can shorten preparation time or simplify the preparation process, the preparation of material library will become more convenient. Fortunately, ordinary color inkjet printers can also be used for enzyme activity verification [18] and multi-component catalyst screening experiments in the biological field [19]. The ink channel of the printer was filled with different materials solution, and then printed out according to the designed template, so as to achieve the purpose of large-scale material preparation. However, in previous research, there was no effective method to match the relationship between the designed inkjet quantity and the actual inkjet quantity [20].

Here, based on the above problems of BPLC and the characteristics of high-throughput technology, this paper attempts to apply high-throughput technology to BPLC for the first time, and proposes a suitable method for high-throughput preparation and detection of BPLC. Fortunately, we finally successfully prepared 1080 BPLC samples in common five-component systems by inkjet printing with high throughput, and developed a high-throughput recognition system for BPLC based on machine learning, and screened out a BPLC material with a relatively wide temperature range. Although there are still some experimental errors and inconvenience, the results are helpful to improve the research efficiency of BP materials and provide a theoretical basis and practical guidance for rapid screening of multi-component BPLC materials.

## 2. High-Throughput Preparation and Detection of BPLC

### 2.1. Materials and Equipment

**Figure 1:**
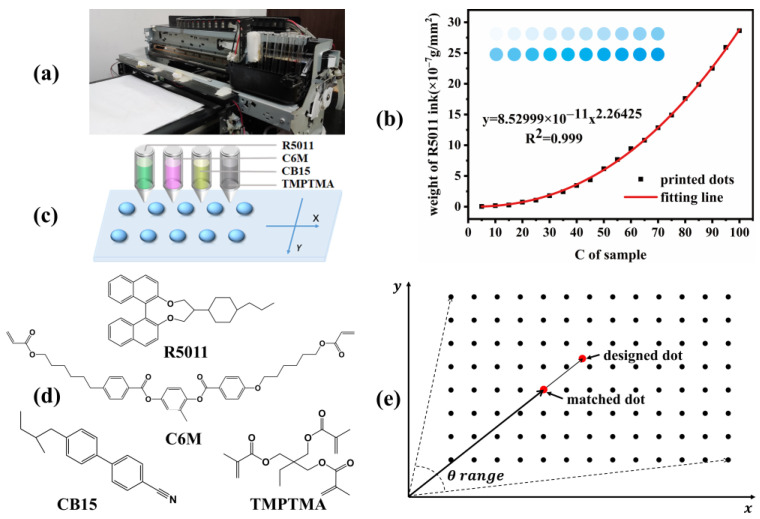
(**a**,**c**) Schematic diagram of inkjet printer device and printing principle. (**b**) Standard curve of 5 wt% R5011 solution for inkjet printing in C channel. (**d**) The molecular structure of the inkjet printing component used. (**e**) Schematic diagram of vector matching method.

In this study, the studied LC sample consists of 5 materials, which are nematic LC matrix (BHR32100, Δn = 0.235, Δε = +30.2, T_N-I_ = 98.0 °C, Beijing BaYi Space LCD Technology Co., Ltd.), 1,4-di-[4-(6-acryloyloxy) hexyloxy benzoyloxy]-2-methyl benzene (C6M, Beijing Kexin Jingyuan Science and Trade Co., Ltd., Beijing, China) and trimethylol propane trimethylmethacrylate (TMPTMA) as monomer, R5011 (JiangSu HeCheng Display Technology Co., Ltd., Nanjing, China, HTP = 120 μm^−1^) and chiral 4-2-methylbutyl-4-cyanobiphenyl (CB15, Beijing BaYi Space LCD Technology Co., Ltd., Beijing, China) as chiral dopant as Figure 1d shows. In addition, polytetrafluoroethylene preparation (PTFE, Dongguan Fuwang New Material Co., Ltd., Dongguan, China) was selected as surface treatment agent, and 2,2-dimethopxy-1,2-diphenyl-ethanone (IRG651, TCI Co., Ltd., Taipei City, Taiwan) was used as photo-initiator. All the Chemical reagents and organic solvents (ethacanol, cyclohexanone) were commercially available and used without further purification. The surface modification of the printed glass substrate is as follows: the glass substrate is cut into 25 × 30 cm^2^, then the glass substrate is placed on the homogenizer, spin coating it with polytetrafluoroethylene /ethanol (1:1) solution, and finally baking it at 180 °C for 30 min to obtain a polytetrafluoroethylene (PTFE)-modified substrate.

Crossed polarizing optical microscope (Olympus BX51) was used to observe and confirm the textures of mesogenic phase, which of each sample were taken by the onboard camera driven by Linksys software (version 2.43, Cao Yingwei, Irvine, CA, USA). The contact angle of the substrate was mainly used to analyze the appropriate substrate for inkjet printing, and measured on the contact angle system (OCA 15, DataPhysics Instruments GmbH, Filderstadt, Germany). A piezoelectric drop-on-demand (DOD) inkjet printer (OS-A3UV-05, Dongsheng, Ltd, Shenzhen, China) was used in high-throughput inkjet printing LC samples, its print head contains six separate ink channels (CMYKWW) which can be controlled independently by software (Acro-rip) and linked to the printed images in CMYK mode. The ink cartridges of the five channels (C, M, Y, K, W) in the printer were, respectively, loaded with cyclohexanone solutions of 5 wt% R5011, 5 wt% C6M, 20 wt% CB15, 5 wt% TMPTMA and 40 wt% BHR32100-100 in sequence. The mixed solution of the corresponding proportion of each component can be printed by controlling the color of the printing pattern. The physical photo and schematic diagram of the inkjet printer are shown in Figure 1a,c).

**Figure 2:**
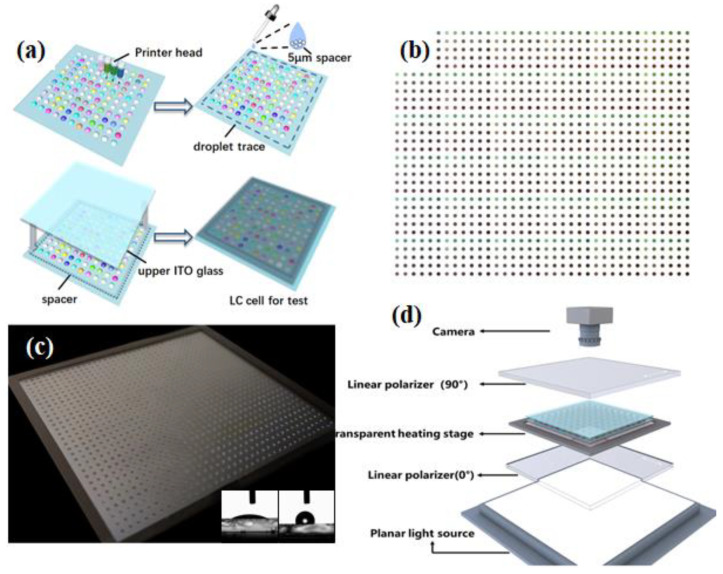
(**a**) Assembly process of liquid crystal high-throughput detection cell. (**b**) CMYK mixed color patterns of 1080 sample points after vector cosine similarity search. (**c**) The 1080 sample point photos were prepared by inkjet printing with high throughput. The inner are the contact angle photos of the glass substrate before and after the surface treatment. (**d**) Device structure for high-throughput detection of LC samples.

Similar to the single LC sample detection device reported in the literature [21], the BPLC high-throughput detection device used is self-made in the laboratory, which is mainly composed of a camera, a transparent hot table with accurate temperature control, a crossed polarizer, a halogen plane light source, and a square wave voltage device (0–200 V), as shown in Figure 2. The programming software used is mainly Labview and Python 3.7, as well as function modules related to image and data processing.

### 2.2. High-Throughput Preparation of BPLC

A comprehensive experiment would be carried out in the host LC BHR32100-100 by high-throughput, mainly to investigate the influence of R5011, C6M, CB15 and TMPTMA on the BP temperature range of mixed liquid crystal. According to the results of previous studies, the value levels of various influencing factors are shown in Table 1. There are 1080 combinations among various factors (6 × 5 × 6 × 6 = 1080) for the above components doping into the host LC, which can be used for high-throughput printing of all BPLC samples, as shown in Table S1 as supplementary information.

How to achieve rapid on-demand mixing between different components of samples is the key to achieve high-throughput preparation and detection in this paper. Fortunately, it is well known that the color can be composed of four basic colors of CMYK, namely C (cyan), M (magenta), Y (yellow), K (black), which are mixed with different chromaticity value of C, M, Y and K, and the chromaticity value of each color channel is between 0 and 100. As we know, CMYK mode is one of the basic modes for color printing of inkjet printers. The CMYK chromaticity value also directly controls the volume of ink ejected from the nozzle in each channel, and then deposits it on the substrate to mix various inks in a certain proportion. Similarly, the mixing ratio of various solutions in each channel on the substrate can be controlled by controlling different CMYK values. In theory, by controlling different CMYK values, the mixing ratio of various solutions in each channel on the substrate during inkjet printing can be controlled, so as to achieve rapid mixing of the components of the sample.

However, the volume or weight of ink ejected from the nozzle is positively correlated with the CMYK chromaticity value of the picture color, but it is not necessarily linearly correlated. Moreover, due to different physical and chemical properties of different inks or solutions, the correlation is not the same. Therefore, in order to accurately control the ink jet amount and obtain relatively accurate proportional components at the LC sample points, we propose a “standard curve method”, that is, to establish the relationship between the chromaticity value (C, M, Y, K, W) of each channel and the deposition weight of the corresponding solution during the printing process, so as to control the solute weight in the solution ejected by the nozzle as needed.

The preparation of standard curve is as follows, taking 5 wt% R5011 ink in cyan channel (C channel) as an example. The 20 cyan circular patterns (8 cm in diameter) with different chromaticity values were made based on CMYK mode by using image processing software (such as Photoshop), that is, the chromaticity values of 20 patterns with an interval of 5 were set in channel C: 5, 10, 15, 20, 25, 30, …, 100, while that of other channels were set to 0. At the same time, 20 pieces of square glass (size 10 × 10 cm) were carefully cut from a large piece of glass and labeled by serial number, and then the weight of each glass was weighed and recorded for the first time. Then, these glasses were spliced together in order to be used as the printing substrate, and placed at the position corresponding to the above pattern arranged with 20 different chromaticity value circles. The 5 wt% R5011 cyclohexanone solution was added to the C channel, and then the combined patterns with different chromaticity values of C channel were sequentially inkjet printed on the substrate. The printed glasses were dried in a 60 °C blast oven for 3 h, and then were weighed and recorded again when the solvent volatilizes completely. When the chromaticity value is small (C < 45), the deposition weight is thus too small to be accurately weighed. Therefore, the accurate deposition weight can be obtained by repeated printing for 3 times and then averaged. By dividing the weight difference of each glass before and after printing by the area of the each circle accordingly, the weight of R5011 deposited on the unit area of the substrate corresponding to 20 different chromaticity values could be obtained. Then, the relation curve between the chromaticity value in the range of 0 and 100 and the R5011 deposition weight can be fitted accordingly, as shown in Figure 1b. Similarly, standard curves of cyclohexanone solutions of other components in other channels can be obtained according to the method.

For single-component samples, only one standard curve is required, and the integer value (0–100) on the X axis corresponds to a decimal mass value on the Y axis, so 100 samples with different qualities can be printed. However, for a five-component sample, you need to query five standard curves (R5011, C6M, BHR32100-100, CB15 and TMPTMA), and the printed sample mass proportion will have 100^5^ possible combinations, forming a fairly large five-dimensional mass vector library. As we know, the print chromaticity value is an integer between 0 and 100, so to obtain the chromaticity value combination of the sample, it is necessary to find the closest matching decimal mass combination (mass vector) in the 5-Dimensional vector library according to the mass ratio (sample vector) of each component of the sample, and then index to find the corresponding print chromaticity value combination (chromaticity vector). This method of matching the sample vector and the mass vector to search the chromaticity vector is called vector query method. As we all know, for two n-dimensional vectors $\vec{a}\left(x_{11},x_{12},\ldots ,x_{1n}\right)$ and $\vec{b}\left(x_{21},x_{22},\ldots ,x_{2n}\right)$, the cosine value of the included angle between vectors can be used to measure the similarity between the two, as shown in Figure 1e. The cosine value of the angle between $\vec{a}$ and $\vec{b}$ is:

(1) $$\cos\left(\theta \right)=\frac{\vec{a}\cdot \vec{b}}{\left|\vec{a}\right|\left|\vec{b}\right|}$$

That is:

(2) $$\cos\left(\theta \right)=\frac{\sum_{k=1}^{n}x_{1k}x_{2k}}{\sqrt{\sum_{k=1}^{n}x_{1k}^{2}}\sqrt{\sum_{k=1}^{n}x_{2k}^{2}}}$$

The value range of the included angle cosine is [−1, 1]. The larger the included angle cosine, the smaller the included angle between the two n-dimensional vectors. When the directions of the two vectors coincide (*θ* = 0), the cosine of the angle takes the maximum value of 1.

Therefore, the so-called vector query method here is to use the cosine distance between the sample vector and the mass vector to measure the similarity of the two vector, find the mass vector with the largest cosine value or the smallest intersection angle with the sample vector from the mass library, and then retrieve the corresponding chromaticity vector through the standard curve. Here, for 1080 samples, the process can obtain the mass vector with the minimum angle with the sample matching vector from the mass vector library one by one, and then index to obtain the corresponding print chromaticity values, finally obtaining 1080 chromaticity value combinations. Then, picture processing software (such as CorelDRAW) can be used to fill 1080 circular patterns with the synthesized color one by one, that is, the color synthesized by combining the chromaticity values corresponding to each sample. In this experiment, in order to facilitate subsequent pressing and detection, the circular pattern was designed as a circle with a diameter of 1 mm and a spacing of 2.2 mm, as shown in Figure 2.

After the samples dot pattern was generated, it was loaded into the software system of the printer, and cyclohexanone solutions of 5 wt% R5011, 5 wt% C6M, 20 wt% CB15, 5 wt% TMPTMA and 40 wt% BHR32100-100 were sequentially injected into the five channels (CMYKW) of the printer, so that samples can be prepared on the substrate by inkjet printing with high throughput.

Except for the liquid crystal channel, the solutions in all other channels were cyclohexanone solutions with component concentrations less than 20 wt%, which were far from saturated. Although the component concentration in the W channel reached 40 wt%, the BHR32100 used was a commercial mixture with each component less than 30 wt%, so the cyclohexanone solution of BHR32100 in the W channel had also not yet reached saturation. In addition, the boiling point of organic solvent cyclohexanone was relatively high (155 °C), so each sample point on the substrate was still in the true solution state after inkjet printing and before drying, and the components in the solution could diffuse freely and be fully mixed. After printing and standing for 10 min, the substrate with samples was placed in a 60 °C blast oven to dry for 3 h. After the solvent volatilized completely, 1080 LC sample libraries were prepared at high throughput. In order to facilitate the subsequent high-throughput detection, another clean substrate of the same size was covered on the LC layer and glued to form a LC sample cell. The LC cell was a sandwich structure, with 5 μm beads as spacers, which were placed between glass substrates near the edges.

### 2.3. High-Throughput Detection of the BPLC Temperature Range

As we know, the blue phase is a self-assembled mesophase, which usually occurs in a narrow temperature range between the cholesteric phase and the isotropic phase. The blue phase can reflect circularly polarized light, but it is not affected by the orientation conditions because it is optically isotropic in the macroscopic. Cholesteric phase can also selectively reflect circularly polarized light, and its reflected wavelength band is wider than that of blue phase, but its orientation state is seriously affected by the boundary conditions. The isotropic state neither reflects circular polarized light nor is affected by orientation conditions.

When the isotropic phase, the blue phase and the cholesteric phase are, respectively, placed between the two glasses without orientation under the cross polarizing plate, the isotropic phase will appear black due to the systematic extinction, while the BP will generally appear colored texture, while the non-oriented cholesteric phase will appear light source color due to the focal cone texture. Therefore, based on these three different optical characteristics, the color information of samples in different phase intervals can be collected by the camera and distinguished by the machine. If there is a very large observation area, the camera can measure multiple samples at the same time. Moreover, it can be expected that the smaller the sample points prepared by high-throughput, the more samples can be detected at the same time. In order to facilitate the collection of color change data of samples with temperature change, we developed a computer program based on machine vision and machine learning, which can locate, collect and analyze the image information of all samples.

**Figure 3:**
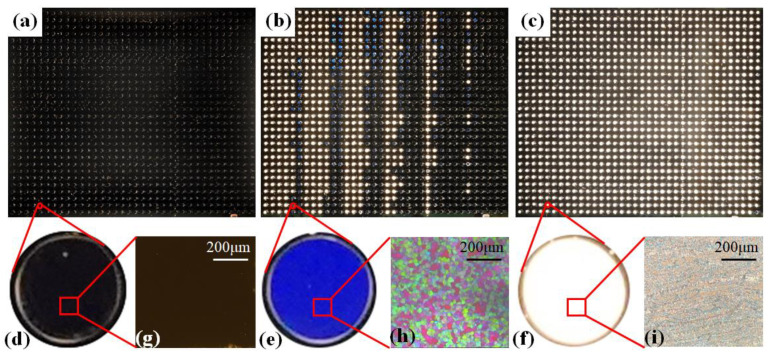
(**a**–**c**) Images of 1080 sample points at the initial (80 °C), middle (39 °C) and final stages (7 °C) of acquisition. (**d**–**f**) The images of sample points in isotropic phase, blue phase and cholesteric phase, respectively, and (**g**–**i**) the corresponding polarizing microscope photos.

The procedure of image data acquisition in this paper is as follows: Firstly, all 1080 samples in the sample test cell were heated to the clear points (e.g., 80 °C), and then were slowly cooled down (0.5 °C/min) after all the samples were clear. The image information of the samples at each temperature was recorded every time the temperature was dropped by 0.5 °C and then maintained for another 1 min to minimize the temperature difference in the substrate. Figure 3a shows when the sample was heated to an isotropic state, all the samples appeared black because the sample did not change the polarization direction of the transmitted light. With the decrease in temperature, some of 1080 samples appeared colorful colors and some appear white, as shown in Figure 3b. It can be confirmed that the colored sample belonged to the blue phase and the white sample was in the cholesteric phase by polarized light microscopy. The substrate was further cooled until the temperature was lower than the upper limit of the cholesteric temperature range of all samples (e.g., 7 °C), and all samples would turn into cholesteric phase, showing the color of the light source, as shown in Figure 3c.

In the whole process of image acquisition, if one photo was collected after cooling down by 0.5 °C, a total of 147 pictures needed to be collected, and each picture contained 1080 sample points, so there were as many as 158,760 sample images of different temperatures that needed to be identified. Because the amount of data was too large to be detected manually, we designed a machine learning algorithm based on convolutional neural network to recognize and classify the phase states of LC sample points. Firstly, the collected images of some samples with known phase states were intercepted as the region of interest (ROI) of the picture, all of which were made as 30 × 30 pixel images. A total of about 1000 intercepted images for each phase category were prepared and divided into training set and test set according to the ratio of 9:1. The data processing module in Python was used to extract the RGB information of the images in the data set, which was then sent to the neural network model built by Tensorflow framework. After multi-layer training and learning, the neural network model for recognizing BPLCs was finally obtained. The overall recognition accuracy of the neural network model to 158,760 sample images was over 93%, which showed that the model was stable and suitable for rapid screening of blue phase.

Through the identification model, we can obtain the phase states of the sample points at different temperatures, that is, the phase transition temperatures of each sample point, including the BP temperature range and the clear point data. Of course, there may be a certain deviation (about 2–3 degrees) between the data obtained by high-throughput and the results obtained by manual mixing and detection by POM observation, but it was still within the acceptable error range, which was fully applicable to rapid screening of a large number of samples in the early stage of blue-phase material research. As for the error, it can mainly be attributed to the reason that the collected image was distorted due to the uneven brightness of the plane light source, and the error in phase recognition was caused by the fact that the sample may have a blue phase III without any texture, or a sphere phase with a similar mosaic texture. Therefore, in this case, additional manual inspection and error correction were required. However, in general, the efficiency of this paper based on machine vision and machine learning methods in high-throughput detection of 1080 samples of blue phase was 100-fold higher than that of manual observation under a polarizing microscope, which can greatly improve the research and development efficiency of blue-phase liquid crystal materials.

### 2.4. Results and Discussion

**Figure 4:**
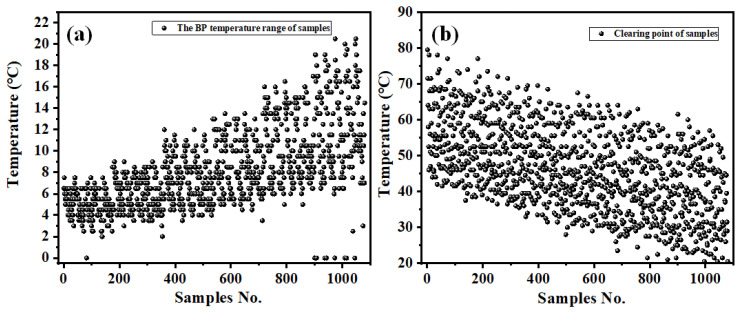
Scatter plot of (**a**) the blue-phase (including spherical phase) temperature range and (**b**) the clear points of 1080 LC samples.

After completing the high-throughput preparation of BPLC sample points and the high-throughput extraction of phase-transition data, we obtained the BP temperature range and clear points of 1080 samples with different ratios. Figure 4 is the scatter plot of the BP temperature range and clear point of 1080 sample points. It could be seen that the BP temperature range of all samples was lower than 20 °C, and some samples did not show blue phase in the whole test process. Among the BP samples, a few samples had a wide BP temperature range of 16~20 °C, and the average BP temperature range was 7.5 °C. The clear point of all samples was within 100 °C, and the average clearness point was 70 °C, while the clearness point of a few samples decreased to 45~50 °C.

**Figure 5:**
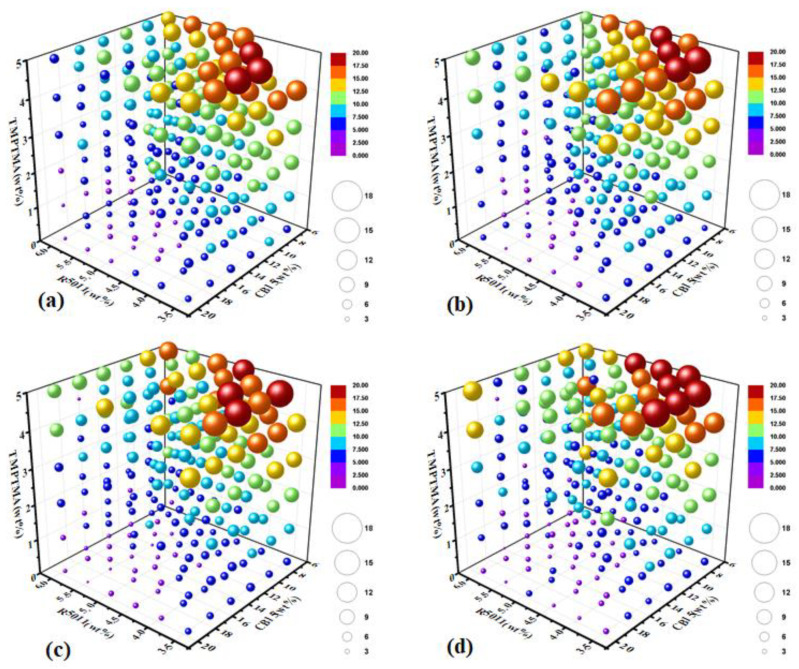
(**a**–**d**) Four-dimensional graph of the blue-phase (including spherical phase) temperature range with different C6M doping amount (0 wt%, 5 wt%, 7.5 wt% and 10 wt%) taking the doping amount of R5011, CB15 and TMPTMA as the coordinate axis.

In order to understand the influence of each component on the BP temperature range, we correspondingly constructed a four-dimensional graph, taking the mass fraction of R5011, CB15 and TMPTMA in the host LC as the X, Y and Z axes of the three-dimensional coordinates, respectively, and using the size or color of the sphere to represent the BP temperature range, as shown in Figure 5. In order to investigate the effect of another component (C6M) in the sample, four-dimensional graphs were correspondingly constructed when the mass fraction of C6M was 0 wt%, 5 wt%, 7.5 wt% and 10 wt%, respectively, and the effects of R5011, CB15 and TMPTMA on the BP temperature range can be clearly observed.

It can be found that the three-dimensional diagrams of different C6M contents had certain similarity, indicating that C6M has a certain effect on the BP temperature range. When the content of C6M was fixed, R5011, CB15 and TMPTMA had different positive effects on the BP temperature range in a certain range. As we know, R5011 and CB15 are chiral compounds, which can increase the chiral content of the system after being doped into the host LC, and thus can effectively play the role of inducing and broadening the blue phase. However, CB15 is a liquid at room temperature and has a very small chiral twisting force, so the addition of excessive CB15 would cause the BP range in the mixed system to move down seriously, that is, the clear point will decrease significantly and the sample will lose the blue phase. Therefore, although CB15 can be used to adjust the upper limit of the BP temperature range, it is still not appropriate to doping too much in the LC system. In addition, because R5011 has a large chiral distortion force, it only needs a small doping amount (3~5 wt%) to achieve the chiral condition of inducing the blue phase without affecting the physical parameters and mesgenic properties of the host LC, so R5011 is often necessary in the BP system.

**Figure 6:**
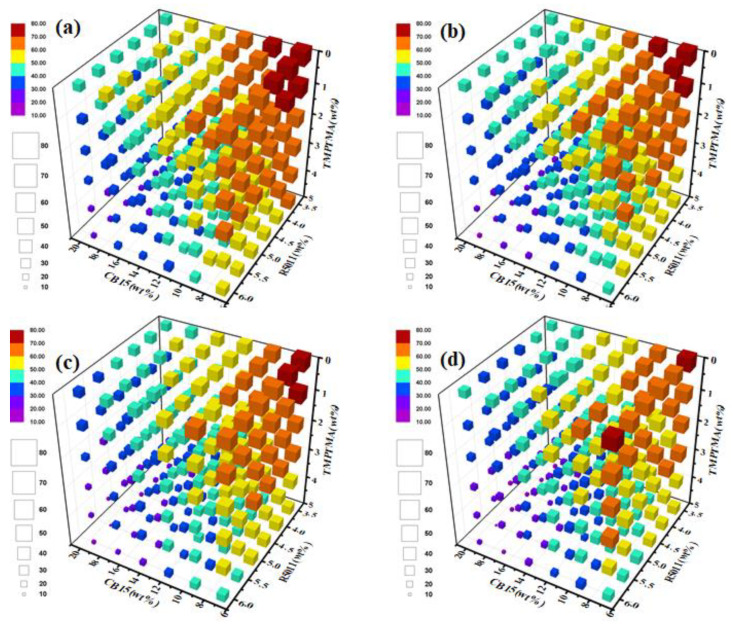
(**a**–**d**) Four-dimensional graph of the clear points with different C6M doping amount (0 wt%, 5 wt%, 7.5 wt% and 10 wt%) taking the doping amount of R5011, CB15 and TMPTMA as the coordinate axis.

The machine recognition model in this paper can not only detect the blue phase, but also identify the cholesteric phase and isotropic state. Accordingly, a four-dimensional map was constructed, taking the mass fractions of R5011, CB15 and TMPTMA in the matrix LC as the X, y and Z axes of the three-dimensional coordinates, respectively, and using the size or color of the sphere to represent the bright points, as shown in Figure 6. It can be seen that the three-dimensional diagrams of different C6M contents were very similar, which showed that C6M had little effect on the clear point of the sample. It can be seen from the figure that R5011, CB15 and TMPTMA had different effects on the clearing point, and the relatively low clearing point temperature appeared in the samples with higher content of R5011, CB15 and TMPTMA components. The effect of CB15 and TMPTMA on the clear point of LC was obviously greater than that of R5011, and CB15 had the largest effect. This was mainly because CB15 and TMPTMA are liquid at room temperature, that is, they can significantly reduce the clear point of the sample. Although R5011 was a solid at room temperature, its melting point was lower than the clear point of the host LC, so the clear point of the mixed system will be reduced to a certain extent after doping. C6M was a mesogenic monomer, which was relatively close to the clear point of the host LC, so it had little influence on the LC parameters of the mixed LC system.

## 3. Conclusions

Based on common inkjet printing and machine vision technology, we successfully developed inkjet printing equipment for high-throughput preparation of LC materials and detection equipment for high-throughput identification of BPLCs. In this paper, 1080 LC samples were designed based on the comprehensive experimental method for high-throughput preparation and screening. By establishing the “standard curves” of BHR32100-100, C6M, R5011, CB15 and TMPTMA components and using the “vector matching method”, the corresponding and accurate chromaticity values of each channel of 1080 sample points for inkjet printing were successfully found in 100^5^ mass vector libraries, Finally, 1080 LC samples were successfully prepared on the substrate with high throughput according to the overall experimental design ratio by using inkjet printing technology. In this paper, based on the method of machine vision, we collected 147 images of 1080 samples packaged in sandwich structure test boxes at different temperatures during cooling. In order to quickly recognize the phase image information of 158,760 sample points, we trained a machine learning model based on convolutional neural network to recognize the mesogenic phase, with an overall accuracy of more than 93%. Compared with the traditional manual detection method, the detection method in this paper can improve the efficiency of BPLC detection by more than 100 fold. By investigating the four-dimensional graph data of BHR32100-100, C6M, R5011, CB15 and TMPTMA on the influence of the BP temperature range and clearing point, it can be found that R5011 and CB15, as chiral compounds, have a positive effect on the induction and stabilization of the BP temperature range in a certain range. Interestingly, TMPTMA, as a nonlinear monomer, also had a positive effect on stabilizing the blue phase. However, CB15 and TMPTMA can significantly reduce the clearing point as well as the phase transition temperature, so it is not appropriate to add too much to LC mixtures. As a mesogenic polymerizable monomer, C6M has little effect on the BP temperature and phase transition temperature before polymerization. These data provide a theoretical basis for further design and development of BPLC components. Although the high-throughput preparation and detection equipment used in this paper also has some problems that need to be further solved, such as the nozzle is easy to block, and the BP recognition rate fails to reach 100%, the high-throughput preparation method and high-throughput detection method in this paper can help to screen the BPLC materials with high throughput, thereby helping to improve the research efficiency of BPLC materials, and thus promoting the practical application of BPLC materials.

## Figures and Tables

**Table 1:** Factor numbers and level numbers of experimental design.

R5011 (wt%)	C6M (wt%)	CB15 (wt%)	TMPTMA (wt%)
3.5	0	7	0
4	2.5	9.5	1
4.5	5	12	2
5	7.5	14.5	3
5.5	10	17	4
6	/	19.5	5

## Data Availability

Not applicable.

## Supplementary Materials

The following supporting information can be downloaded at: https://www.mdpi.com/article/10.3390/molecules27206938/s1, Figure S1: Merged Patterns of Circles (8 cm in Diameter) with Different Chroma Values; Table S1: Deposition weight of 5 wt% R5011 cyclohexanone solution for inkjet printing at different chromaticity values; Figure S2: The standard curves corresponding to the cyclohexanone solution of 5 wt% R5011, 5 wt% C6M, 40 wt% BHR32100, 20 wt% CB1 and 5 wt% TMPTMA are respectively inkjet printed in the CMWYK channel. Table S2: Composition and proportion of liquid crystal samples for high-throughput ink-jet printing

## Sample Availability

Samples of the compounds are not available from the authors.
