# Supplementary material for: High-Throughput Preparation and Machine Learning Screening of a Blue-Phase Liquid Crystal Based on Inkjet Printing
Source: Molecules. 2022 Oct 16;27(20):6938. doi: 10.3390/molecules27206938 (PMC9608808; doi:10.3390/molecules27206938)
Supplement: Supplementary file 1 [file molecules-27-06938-s001.zip › molecules-1952374-supplementary.pdf]

Electronic Supplementary Information

High Throughput Preparation and Machine Learning Screening of Blue Phase Liquid Crystal Based on Inkjet Printing

WanLi He*#, YongFeng Cui#, ShiGuang Luo#, WenTuo Hu, KaiNan Wang, Zhou Yang, Hui Cao, Dong Wang

1. Standard curves of 5wt% R5011, 5wt% C6M, 40wt% BHR32100, 20wt% CB15 and 5wt% TMPTMA solutions in CMWYK channels

The preparation of standard curve is as follows: Taking 5wt% R5011 ink in cyan channel (C channel) as an example. 20 cyan circular patterns (8cm in diameter) with different chromaticity values were made based on CMYK mode by using image processing software (such as Photoshop), that is, the chromaticity values of 20 patterns with an interval of 5 were set in channel C: 5, 10, 15, 20, 25, 30,..., 100, while that of other channels were set to 0, as shown in **Figure S1**.

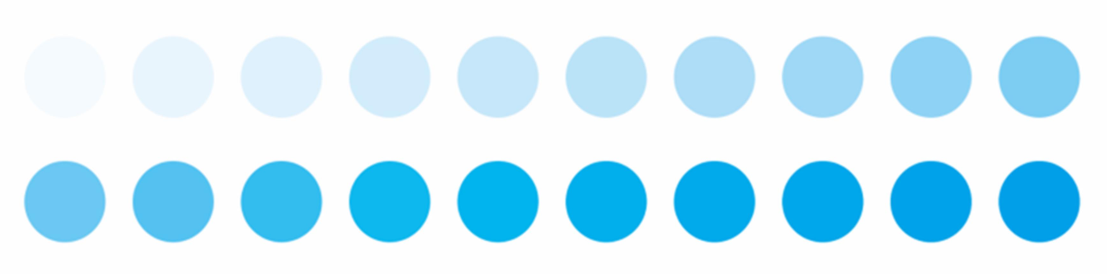

Figure S1. Merged Patterns of Circles (8cm in Diameter) with Different Chroma Values

At the same time, 20 pieces of square glass (size 10 × 10 cm) were carefully cut from a large piece of glass and labeled by serial number, and then the weight of each glass was weighed and recorded for the first time. Then, these glasses were spliced together in order to be used as the printing substrate, and placed at the position corresponding to the above pattern arranged with 20 different chromaticity value circles. 5wt% R5011 cyclohexanone solution was added to the C channel, and then the combined patterns with different chromaticity values of C channel were sequentially inkjet printed on the substrate. The printed glasses were dried in a 60 ℃ blast oven for 3 hours, and then were weighed and recorded again when the solvent

volatilizes completely. When the chromaticity value is small (C<45), the deposition weight is thus too small to be accurately weighed. Therefore, the accurate deposition weight can be obtained by repeated printing for 3 times and then averaged. By dividing the weight difference of each glass before and after printing by the area of the each circle accordingly, the weight of R5011 deposited on the unit area of the substrate corresponding to 20 different chromaticity values could be obtained, as shown in **Table S1**. Then the relation curve between the chromaticity value in the range of 0 and 100 and the R5011 deposition weight can be fitted accordingly. Similarly, standard curves of cyclohexanone solutions of other components in other channels can be obtained according to the method, as shown in **Figure S2**.

Table S1. Deposition weight of 5 wt% R5011 cyclohexanone solution for inkjet printing at different chromaticity values

C value	Glass weight before printing(g)	Glass weight after printing(g)	Deposition weight of R5011 (g)	Area of circle/mm2	Unit Deposition weight of R5011 (g)
5	10.71803833	10.71805833	2E-05	5024	3.98089E-09
10	11.165245	11.16533	8.5E-05	5024	1.69188E-08
15	10.77425333	10.77439167	0.000138333	5024	2.75345E-08
20	10.88630833	10.88667833	0.00037	5024	7.36465E-08
25	10.85176167	10.85229833	0.000536667	5024	1.06821E-07
30	10.99565667	10.99655667	0.0009	5024	1.7914E-07
35	10.88239	10.88361167	0.001221667	5024	2.43166E-07
40	11.03692333	11.03866167	0.001738333	5024	3.46006E-07
45	33.826025	33.82822	0.002195	5024	4.36903E-07
50	33.96233	33.965425	0.003095	5024	6.16043E-07
55	33.4477	33.45155	0.00385	5024	7.66322E-07
60	33.02119	33.02593	0.00474	5024	9.43471E-07
65	32.962445	32.967875	0.00543	5024	1.08081E-06
70	32.69404	32.70049	0.00645	5024	1.28384E-06
75	32.278465	32.28595	0.007485	5024	1.48985E-06
80	32.615395	32.624235	0.00884	5024	1.75955E-06
85	33.100795	33.11078	0.009985	5024	1.98746E-06
90	32.644835	32.65614	0.011305	5024	2.2502E-06
95	33.71531	33.728325	0.013015	5024	2.59057E-06
100	33.122245	33.136625	0.01438	5024	2.86226E-06

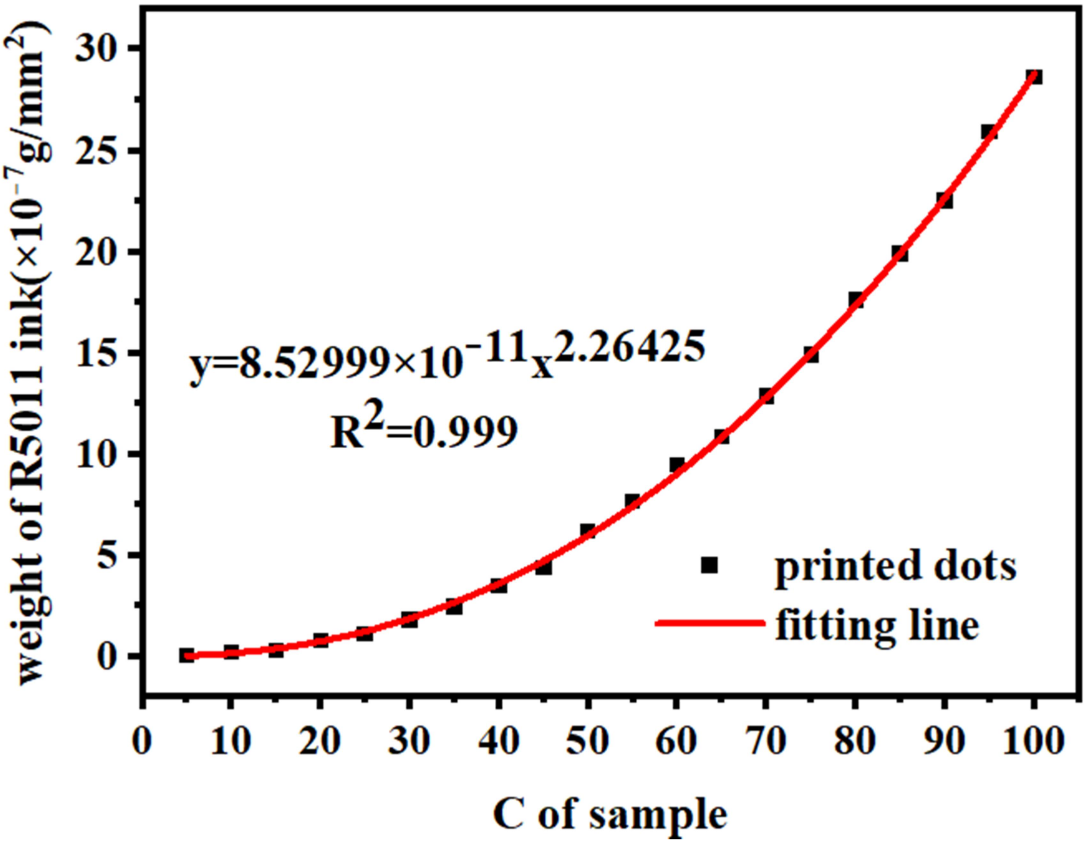

weight of R5011 ink(×10⁻⁷g/mm²)
y=8.52999×10⁻¹¹x^2.26425
R²=0.999
printed dots
fitting line
C of sample

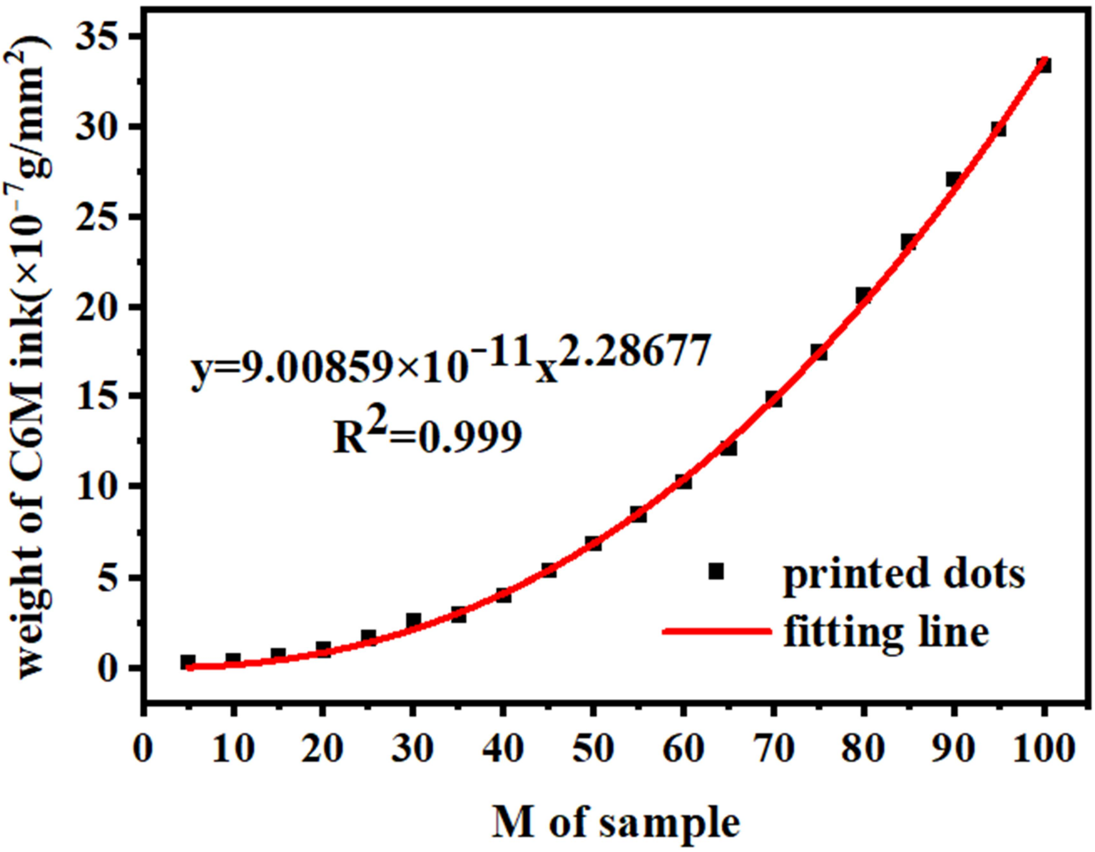

weight of C6M ink(×10⁻⁷g/mm²)
y=9.00859×10⁻¹¹x^2.28677
R²=0.999
printed dots
fitting line
M of sample

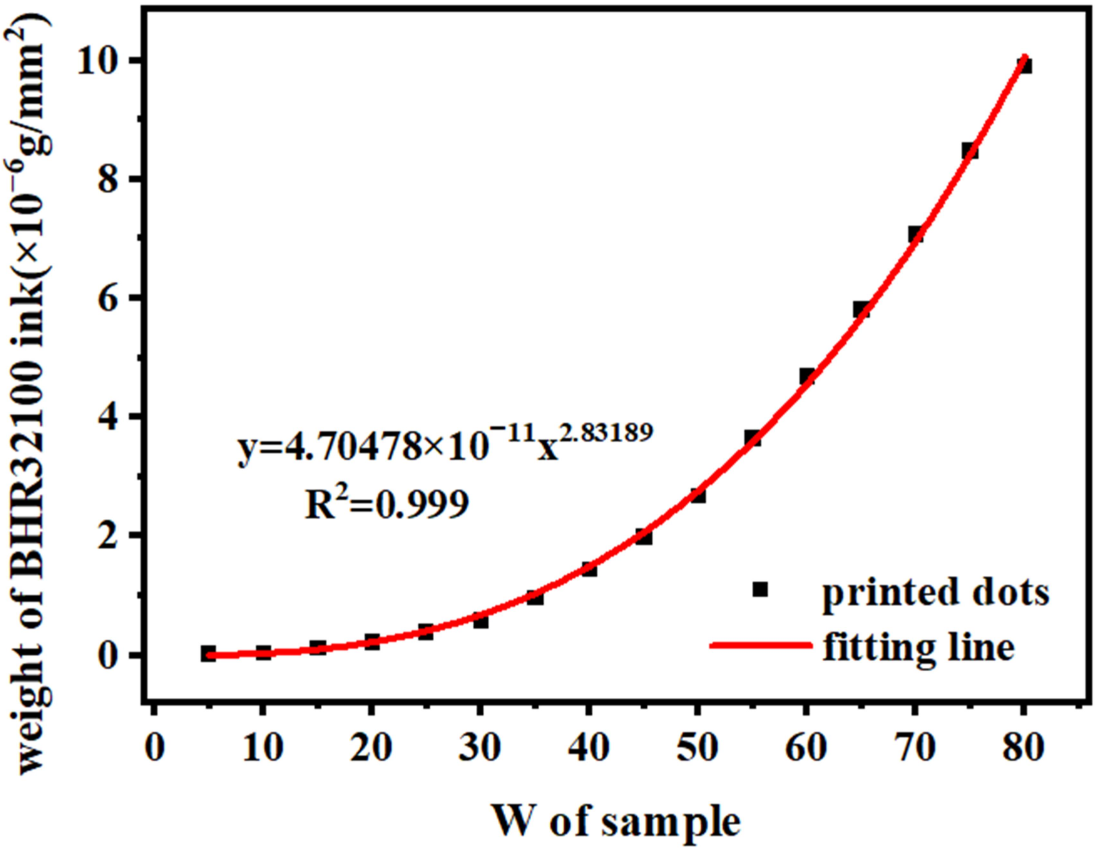

weight of BHR32100 ink(×10⁻⁶g/mm²)
W of sample
y=4.70478×10⁻¹¹x^2.83189
R²=0.999
printed dots
fitting line

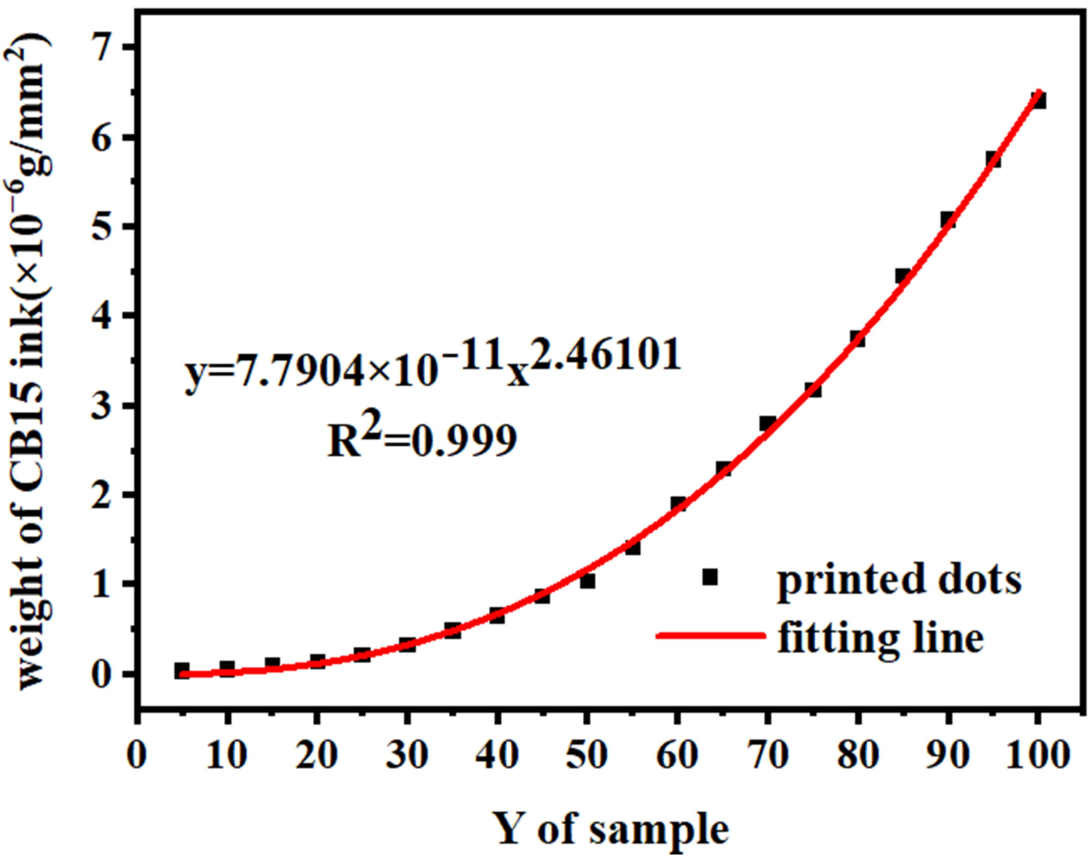

weight of CB15 ink(×10⁻⁶g/mm²)
Y of sample
y=7.7904×10⁻¹¹x^2.46101
R²=0.999
printed dots
fitting line

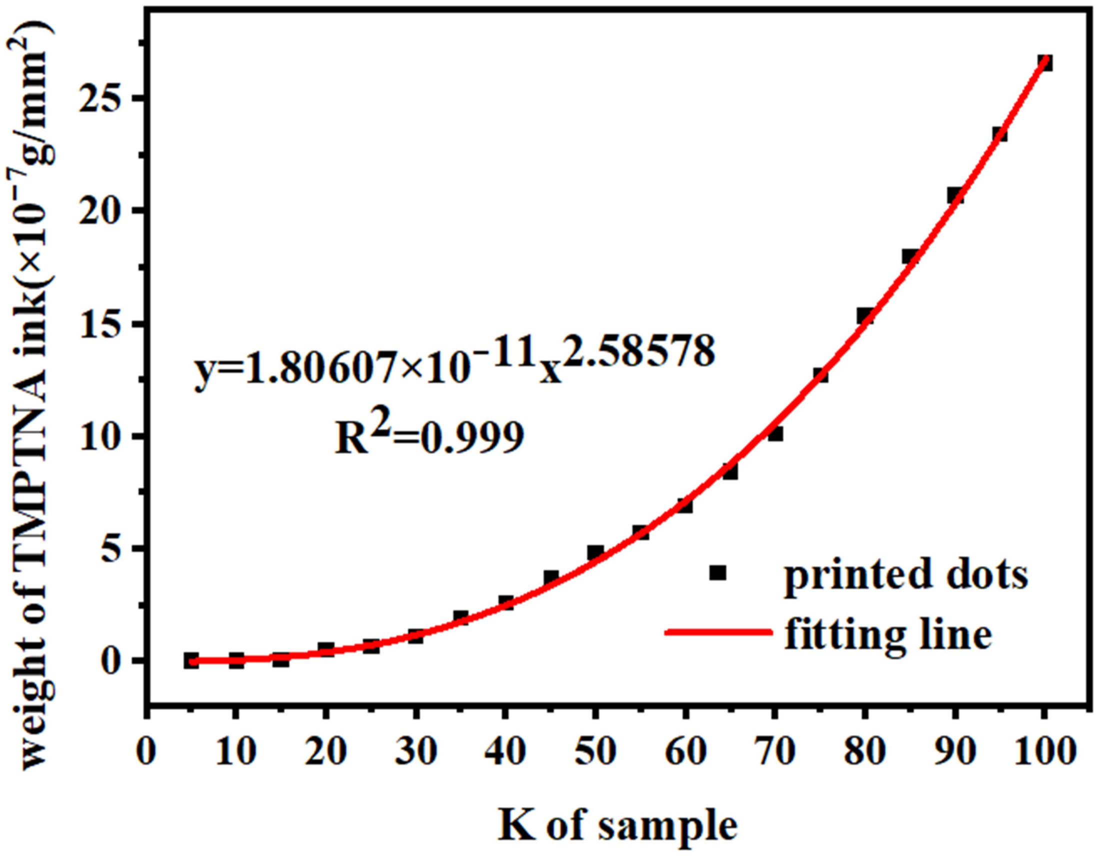

Figure S2. The standard curves corresponding to the cyclohexanone solution of 5wt% R5011, 5wt% C6M, 40wt% BHR32100, 20wt% CB1 and 5wt% TMPTMA are respectively inkjet printed in the CMWYK channel

Table S2 . Composition and proportion of liquid crystal samples for high-throughput ink-jet printing

Sample No	TMPTMA (wt%)	CB15 (wt%)	C6M (wt%)	R5011 (wt%)	BHR32100 (wt%)
1	0	7	0	3.5	89.5
2	0	9.5	0	3.5	87
3	0	12	0	3.5	84.5
4	0	14.5	0	3.5	82
5	0	17	0	3.5	79.5
6	0	19.5	0	3.5	77
7	0	7	0	4	89
8	0	9.5	0	4	86.5
9	0	12	0	4	84
10	0	14.5	0	4	81.5

11	0	17	0	4	79
12	0	19.5	0	4	76.5
13	0	7	0	4.5	88.5
14	0	9.5	0	4.5	86
15	0	12	0	4.5	83.5
16	0	14.5	0	4.5	81
17	0	17	0	4.5	78.5
18	0	19.5	0	4.5	76
19	0	7	0	5	88
20	0	9.5	0	5	85.5
21	0	12	0	5	83
22	0	14.5	0	5	80.5
23	0	17	0	5	78
24	0	19.5	0	5	75.5
25	0	7	0	5.5	87.5
26	0	9.5	0	5.5	85
27	0	12	0	5.5	82.5
28	0	14.5	0	5.5	80
29	0	17	0	5.5	77.5
30	0	19.5	0	5.5	75
31	0	7	0	6	87
32	0	9.5	0	6	84.5
33	0	12	0	6	82
34	0	14.5	0	6	79.5
35	0	17	0	6	77
36	0	19.5	0	6	74.5
37	0	7	2.5	3.5	87
38	0	9.5	2.5	3.5	84.5
39	0	12	2.5	3.5	82
40	0	14.5	2.5	3.5	79.5
41	0	17	2.5	3.5	77
42	0	19.5	2.5	3.5	74.5
43	0	7	2.5	4	86.5
44	0	9.5	2.5	4	84
45	0	12	2.5	4	81.5
46	0	14.5	2.5	4	79
47	0	17	2.5	4	76.5
48	0	19.5	2.5	4	74
49	0	7	2.5	4.5	86
50	0	9.5	2.5	4.5	83.5
51	0	12	2.5	4.5	81
52	0	14.5	2.5	4.5	78.5
53	0	17	2.5	4.5	76
54	0	19.5	2.5	4.5	73.5

55	0	7	2.5	5	85.5
56	0	9.5	2.5	5	83
57	0	12	2.5	5	80.5
58	0	14.5	2.5	5	78
59	0	17	2.5	5	75.5
60	0	19.5	2.5	5	73
61	0	7	2.5	5.5	85
62	0	9.5	2.5	5.5	82.5
63	0	12	2.5	5.5	80
64	0	14.5	2.5	5.5	77.5
65	0	17	2.5	5.5	75
66	0	19.5	2.5	5.5	72.5
67	0	7	2.5	6	84.5
68	0	9.5	2.5	6	82
69	0	12	2.5	6	79.5
70	0	14.5	2.5	6	77
71	0	17	2.5	6	74.5
72	0	19.5	2.5	6	72
73	0	7	5	3.5	84.5
74	0	9.5	5	3.5	82
75	0	12	5	3.5	79.5
76	0	14.5	5	3.5	77
77	0	17	5	3.5	74.5
78	0	19.5	5	3.5	72
79	0	7	5	4	84
80	0	9.5	5	4	81.5
81	0	12	5	4	79
82	0	14.5	5	4	76.5
83	0	17	5	4	74
84	0	19.5	5	4	71.5
85	0	7	5	4.5	83.5
86	0	9.5	5	4.5	81
87	0	12	5	4.5	78.5
88	0	14.5	5	4.5	76
89	0	17	5	4.5	73.5
90	0	19.5	5	4.5	71
91	0	7	5	5	83
92	0	9.5	5	5	80.5
93	0	12	5	5	78
94	0	14.5	5	5	75.5
95	0	17	5	5	73
96	0	19.5	5	5	70.5
97	0	7	5	5.5	82.5
98	0	9.5	5	5.5	80

99	0	12	5	5.5	77.5
100	0	14.5	5	5.5	75
101	0	17	5	5.5	72.5
102	0	19.5	5	5.5	70
103	0	7	5	6	82
104	0	9.5	5	6	79.5
105	0	12	5	6	77
106	0	14.5	5	6	74.5
107	0	17	5	6	72
108	0	19.5	5	6	69.5
109	0	7	7.5	3.5	82
110	0	9.5	7.5	3.5	79.5
111	0	12	7.5	3.5	77
112	0	14.5	7.5	3.5	74.5
113	0	17	7.5	3.5	72
114	0	19.5	7.5	3.5	69.5
115	0	7	7.5	4	81.5
116	0	9.5	7.5	4	79
117	0	12	7.5	4	76.5
118	0	14.5	7.5	4	74
119	0	17	7.5	4	71.5
120	0	19.5	7.5	4	69
121	0	7	7.5	4.5	81
122	0	9.5	7.5	4.5	78.5
123	0	12	7.5	4.5	76
124	0	14.5	7.5	4.5	73.5
125	0	17	7.5	4.5	71
126	0	19.5	7.5	4.5	68.5
127	0	7	7.5	5	80.5
128	0	9.5	7.5	5	78
129	0	12	7.5	5	75.5
130	0	14.5	7.5	5	73
131	0	17	7.5	5	70.5
132	0	19.5	7.5	5	68
133	0	7	7.5	5.5	80
134	0	9.5	7.5	5.5	77.5
135	0	12	7.5	5.5	75
136	0	14.5	7.5	5.5	72.5
137	0	17	7.5	5.5	70
138	0	19.5	7.5	5.5	67.5
139	0	7	7.5	6	79.5
140	0	9.5	7.5	6	77
141	0	12	7.5	6	74.5
142	0	14.5	7.5	6	72

143	0	17	7.5	6	69.5
144	0	19.5	7.5	6	67
145	0	7	10	3.5	79.5
146	0	9.5	10	3.5	77
147	0	12	10	3.5	74.5
148	0	14.5	10	3.5	72
149	0	17	10	3.5	69.5
150	0	19.5	10	3.5	67
151	0	7	10	4	79
152	0	9.5	10	4	76.5
153	0	12	10	4	74
154	0	14.5	10	4	71.5
155	0	17	10	4	69
156	0	19.5	10	4	66.5
157	0	7	10	4.5	78.5
158	0	9.5	10	4.5	76
159	0	12	10	4.5	73.5
160	0	14.5	10	4.5	71
161	0	17	10	4.5	68.5
162	0	19.5	10	4.5	66
163	0	7	10	5	78
164	0	9.5	10	5	75.5
165	0	12	10	5	73
166	0	14.5	10	5	70.5
167	0	17	10	5	68
168	0	19.5	10	5	65.5
169	0	7	10	5.5	77.5
170	0	9.5	10	5.5	75
171	0	12	10	5.5	72.5
172	0	14.5	10	5.5	70
173	0	17	10	5.5	67.5
174	0	19.5	10	5.5	65
175	0	7	10	6	77
176	0	9.5	10	6	74.5
177	0	12	10	6	72
178	0	14.5	10	6	69.5
179	0	17	10	6	67
180	0	19.5	10	6	64.5
181	1	7	0	3.5	88.5
182	1	9.5	0	3.5	86
183	1	12	0	3.5	83.5
184	1	14.5	0	3.5	81
185	1	17	0	3.5	78.5
186	1	19.5	0	3.5	76

187	1	7	0	4	88
188	1	9.5	0	4	85.5
189	1	12	0	4	83
190	1	14.5	0	4	80.5
191	1	17	0	4	78
192	1	19.5	0	4	75.5
193	1	7	0	4.5	87.5
194	1	9.5	0	4.5	85
195	1	12	0	4.5	82.5
196	1	14.5	0	4.5	80
197	1	17	0	4.5	77.5
198	1	19.5	0	4.5	75
199	1	7	0	5	87
200	1	9.5	0	5	84.5
201	1	12	0	5	82
202	1	14.5	0	5	79.5
203	1	17	0	5	77
204	1	19.5	0	5	74.5
205	1	7	0	5.5	86.5
206	1	9.5	0	5.5	84
207	1	12	0	5.5	81.5
208	1	14.5	0	5.5	79
209	1	17	0	5.5	76.5
210	1	19.5	0	5.5	74
211	1	7	0	6	86
212	1	9.5	0	6	83.5
213	1	12	0	6	81
214	1	14.5	0	6	78.5
215	1	17	0	6	76
216	1	19.5	0	6	73.5
217	1	7	2.5	3.5	86
218	1	9.5	2.5	3.5	83.5
219	1	12	2.5	3.5	81
220	1	14.5	2.5	3.5	78.5
221	1	17	2.5	3.5	76
222	1	19.5	2.5	3.5	73.5
223	1	7	2.5	4	85.5
224	1	9.5	2.5	4	83
225	1	12	2.5	4	80.5
226	1	14.5	2.5	4	78
227	1	17	2.5	4	75.5
228	1	19.5	2.5	4	73
229	1	7	2.5	4.5	85
230	1	9.5	2.5	4.5	82.5

231	1	12	2.5	4.5	80
232	1	14.5	2.5	4.5	77.5
233	1	17	2.5	4.5	75
234	1	19.5	2.5	4.5	72.5
235	1	7	2.5	5	84.5
236	1	9.5	2.5	5	82
237	1	12	2.5	5	79.5
238	1	14.5	2.5	5	77
239	1	17	2.5	5	74.5
240	1	19.5	2.5	5	72
241	1	7	2.5	5.5	84
242	1	9.5	2.5	5.5	81.5
243	1	12	2.5	5.5	79
244	1	14.5	2.5	5.5	76.5
245	1	17	2.5	5.5	74
246	1	19.5	2.5	5.5	71.5
247	1	7	2.5	6	83.5
248	1	9.5	2.5	6	81
249	1	12	2.5	6	78.5
250	1	14.5	2.5	6	76
251	1	17	2.5	6	73.5
252	1	19.5	2.5	6	71
253	1	7	5	3.5	83.5
254	1	9.5	5	3.5	81
255	1	12	5	3.5	78.5
256	1	14.5	5	3.5	76
257	1	17	5	3.5	73.5
258	1	19.5	5	3.5	71
259	1	7	5	4	83
260	1	9.5	5	4	80.5
261	1	12	5	4	78
262	1	14.5	5	4	75.5
263	1	17	5	4	73
264	1	19.5	5	4	70.5
265	1	7	5	4.5	82.5
266	1	9.5	5	4.5	80
267	1	12	5	4.5	77.5
268	1	14.5	5	4.5	75
269	1	17	5	4.5	72.5
270	1	19.5	5	4.5	70
271	1	7	5	5	82
272	1	9.5	5	5	79.5
273	1	12	5	5	77
274	1	14.5	5	5	74.5

275	1	17	5	5	72
276	1	19.5	5	5	69.5
277	1	7	5	5.5	81.5
278	1	9.5	5	5.5	79
279	1	12	5	5.5	76.5
280	1	14.5	5	5.5	74
281	1	17	5	5.5	71.5
282	1	19.5	5	5.5	69
283	1	7	5	6	81
284	1	9.5	5	6	78.5
285	1	12	5	6	76
286	1	14.5	5	6	73.5
287	1	17	5	6	71
288	1	19.5	5	6	68.5
289	1	7	7.5	3.5	81
290	1	9.5	7.5	3.5	78.5
291	1	12	7.5	3.5	76
292	1	14.5	7.5	3.5	73.5
293	1	17	7.5	3.5	71
294	1	19.5	7.5	3.5	68.5
295	1	7	7.5	4	80.5
296	1	9.5	7.5	4	78
297	1	12	7.5	4	75.5
298	1	14.5	7.5	4	73
299	1	17	7.5	4	70.5
300	1	19.5	7.5	4	68
301	1	7	7.5	4.5	80
302	1	9.5	7.5	4.5	77.5
303	1	12	7.5	4.5	75
304	1	14.5	7.5	4.5	72.5
305	1	17	7.5	4.5	70
306	1	19.5	7.5	4.5	67.5
307	1	7	7.5	5	79.5
308	1	9.5	7.5	5	77
309	1	12	7.5	5	74.5
310	1	14.5	7.5	5	72
311	1	17	7.5	5	69.5
312	1	19.5	7.5	5	67
313	1	7	7.5	5.5	79
314	1	9.5	7.5	5.5	76.5
315	1	12	7.5	5.5	74
316	1	14.5	7.5	5.5	71.5
317	1	17	7.5	5.5	69
318	1	19.5	7.5	5.5	66.5

319	1	7	7.5	6	78.5
320	1	9.5	7.5	6	76
321	1	12	7.5	6	73.5
322	1	14.5	7.5	6	71
323	1	17	7.5	6	68.5
324	1	19.5	7.5	6	66
325	1	7	10	3.5	78.5
326	1	9.5	10	3.5	76
327	1	12	10	3.5	73.5
328	1	14.5	10	3.5	71
329	1	17	10	3.5	68.5
330	1	19.5	10	3.5	66
331	1	7	10	4	78
332	1	9.5	10	4	75.5
333	1	12	10	4	73
334	1	14.5	10	4	70.5
335	1	17	10	4	68
336	1	19.5	10	4	65.5
337	1	7	10	4.5	77.5
338	1	9.5	10	4.5	75
339	1	12	10	4.5	72.5
340	1	14.5	10	4.5	70
341	1	17	10	4.5	67.5
342	1	19.5	10	4.5	65
343	1	7	10	5	77
344	1	9.5	10	5	74.5
345	1	12	10	5	72
346	1	14.5	10	5	69.5
347	1	17	10	5	67
348	1	19.5	10	5	64.5
349	1	7	10	5.5	76.5
350	1	9.5	10	5.5	74
351	1	12	10	5.5	71.5
352	1	14.5	10	5.5	69
353	1	17	10	5.5	66.5
354	1	19.5	10	5.5	64
355	1	7	10	6	76
356	1	9.5	10	6	73.5
357	1	12	10	6	71
358	1	14.5	10	6	68.5
359	1	17	10	6	66
360	1	19.5	10	6	63.5
361	2	7	0	3.5	87.5
362	2	9.5	0	3.5	85

363	2	12	0	3.5	82.5
364	2	14.5	0	3.5	80
365	2	17	0	3.5	77.5
366	2	19.5	0	3.5	75
367	2	7	0	4	87
368	2	9.5	0	4	84.5
369	2	12	0	4	82
370	2	14.5	0	4	79.5
371	2	17	0	4	77
372	2	19.5	0	4	74.5
373	2	7	0	4.5	86.5
374	2	9.5	0	4.5	84
375	2	12	0	4.5	81.5
376	2	14.5	0	4.5	79
377	2	17	0	4.5	76.5
378	2	19.5	0	4.5	74
379	2	7	0	5	86
380	2	9.5	0	5	83.5
381	2	12	0	5	81
382	2	14.5	0	5	78.5
383	2	17	0	5	76
384	2	19.5	0	5	73.5
385	2	7	0	5.5	85.5
386	2	9.5	0	5.5	83
387	2	12	0	5.5	80.5
388	2	14.5	0	5.5	78
389	2	17	0	5.5	75.5
390	2	19.5	0	5.5	73
391	2	7	0	6	85
392	2	9.5	0	6	82.5
393	2	12	0	6	80
394	2	14.5	0	6	77.5
395	2	17	0	6	75
396	2	19.5	0	6	72.5
397	2	7	2.5	3.5	85
398	2	9.5	2.5	3.5	82.5
399	2	12	2.5	3.5	80
400	2	14.5	2.5	3.5	77.5
401	2	17	2.5	3.5	75
402	2	19.5	2.5	3.5	72.5
403	2	7	2.5	4	84.5
404	2	9.5	2.5	4	82
405	2	12	2.5	4	79.5
406	2	14.5	2.5	4	77

407	2	17	2.5	4	74.5
408	2	19.5	2.5	4	72
409	2	7	2.5	4.5	84
410	2	9.5	2.5	4.5	81.5
411	2	12	2.5	4.5	79
412	2	14.5	2.5	4.5	76.5
413	2	17	2.5	4.5	74
414	2	19.5	2.5	4.5	71.5
415	2	7	2.5	5	83.5
416	2	9.5	2.5	5	81
417	2	12	2.5	5	78.5
418	2	14.5	2.5	5	76
419	2	17	2.5	5	73.5
420	2	19.5	2.5	5	71
421	2	7	2.5	5.5	83
422	2	9.5	2.5	5.5	80.5
423	2	12	2.5	5.5	78
424	2	14.5	2.5	5.5	75.5
425	2	17	2.5	5.5	73
426	2	19.5	2.5	5.5	70.5
427	2	7	2.5	6	82.5
428	2	9.5	2.5	6	80
429	2	12	2.5	6	77.5
430	2	14.5	2.5	6	75
431	2	17	2.5	6	72.5
432	2	19.5	2.5	6	70
433	2	7	5	3.5	82.5
434	2	9.5	5	3.5	80
435	2	12	5	3.5	77.5
436	2	14.5	5	3.5	75
437	2	17	5	3.5	72.5
438	2	19.5	5	3.5	70
439	2	7	5	4	82
440	2	9.5	5	4	79.5
441	2	12	5	4	77
442	2	14.5	5	4	74.5
443	2	17	5	4	72
444	2	19.5	5	4	69.5
445	2	7	5	4.5	81.5
446	2	9.5	5	4.5	79
447	2	12	5	4.5	76.5
448	2	14.5	5	4.5	74
449	2	17	5	4.5	71.5
450	2	19.5	5	4.5	69

451	2	7	5	5	81
452	2	9.5	5	5	78.5
453	2	12	5	5	76
454	2	14.5	5	5	73.5
455	2	17	5	5	71
456	2	19.5	5	5	68.5
457	2	7	5	5.5	80.5
458	2	9.5	5	5.5	78
459	2	12	5	5.5	75.5
460	2	14.5	5	5.5	73
461	2	17	5	5.5	70.5
462	2	19.5	5	5.5	68
463	2	7	5	6	80
464	2	9.5	5	6	77.5
465	2	12	5	6	75
466	2	14.5	5	6	72.5
467	2	17	5	6	70
468	2	19.5	5	6	67.5
469	2	7	7.5	3.5	80
470	2	9.5	7.5	3.5	77.5
471	2	12	7.5	3.5	75
472	2	14.5	7.5	3.5	72.5
473	2	17	7.5	3.5	70
474	2	19.5	7.5	3.5	67.5
475	2	7	7.5	4	79.5
476	2	9.5	7.5	4	77
477	2	12	7.5	4	74.5
478	2	14.5	7.5	4	72
479	2	17	7.5	4	69.5
480	2	19.5	7.5	4	67
481	2	7	7.5	4.5	79
482	2	9.5	7.5	4.5	76.5
483	2	12	7.5	4.5	74
484	2	14.5	7.5	4.5	71.5
485	2	17	7.5	4.5	69
486	2	19.5	7.5	4.5	66.5
487	2	7	7.5	5	78.5
488	2	9.5	7.5	5	76
489	2	12	7.5	5	73.5
490	2	14.5	7.5	5	71
491	2	17	7.5	5	68.5
492	2	19.5	7.5	5	66
493	2	7	7.5	5.5	78
494	2	9.5	7.5	5.5	75.5

495	2	12	7.5	5.5	73
496	2	14.5	7.5	5.5	70.5
497	2	17	7.5	5.5	68
498	2	19.5	7.5	5.5	65.5
499	2	7	7.5	6	77.5
500	2	9.5	7.5	6	75
501	2	12	7.5	6	72.5
502	2	14.5	7.5	6	70
503	2	17	7.5	6	67.5
504	2	19.5	7.5	6	65
505	2	7	10	3.5	77.5
506	2	9.5	10	3.5	75
507	2	12	10	3.5	72.5
508	2	14.5	10	3.5	70
509	2	17	10	3.5	67.5
510	2	19.5	10	3.5	65
511	2	7	10	4	77
512	2	9.5	10	4	74.5
513	2	12	10	4	72
514	2	14.5	10	4	69.5
515	2	17	10	4	67
516	2	19.5	10	4	64.5
517	2	7	10	4.5	76.5
518	2	9.5	10	4.5	74
519	2	12	10	4.5	71.5
520	2	14.5	10	4.5	69
521	2	17	10	4.5	66.5
522	2	19.5	10	4.5	64
523	2	7	10	5	76
524	2	9.5	10	5	73.5
525	2	12	10	5	71
526	2	14.5	10	5	68.5
527	2	17	10	5	66
528	2	19.5	10	5	63.5
529	2	7	10	5.5	75.5
530	2	9.5	10	5.5	73
531	2	12	10	5.5	70.5
532	2	14.5	10	5.5	68
533	2	17	10	5.5	65.5
534	2	19.5	10	5.5	63
535	2	7	10	6	75
536	2	9.5	10	6	72.5
537	2	12	10	6	70
538	2	14.5	10	6	67.5

539	2	17	10	6	65
540	2	19.5	10	6	62.5
541	3	7	0	3.5	86.5
542	3	9.5	0	3.5	84
543	3	12	0	3.5	81.5
544	3	14.5	0	3.5	79
545	3	17	0	3.5	76.5
546	3	19.5	0	3.5	74
547	3	7	0	4	86
548	3	9.5	0	4	83.5
549	3	12	0	4	81
550	3	14.5	0	4	78.5
551	3	17	0	4	76
552	3	19.5	0	4	73.5
553	3	7	0	4.5	85.5
554	3	9.5	0	4.5	83
555	3	12	0	4.5	80.5
556	3	14.5	0	4.5	78
557	3	17	0	4.5	75.5
558	3	19.5	0	4.5	73
559	3	7	0	5	85
560	3	9.5	0	5	82.5
561	3	12	0	5	80
562	3	14.5	0	5	77.5
563	3	17	0	5	75
564	3	19.5	0	5	72.5
565	3	7	0	5.5	84.5
566	3	9.5	0	5.5	82
567	3	12	0	5.5	79.5
568	3	14.5	0	5.5	77
569	3	17	0	5.5	74.5
570	3	19.5	0	5.5	72
571	3	7	0	6	84
572	3	9.5	0	6	81.5
573	3	12	0	6	79
574	3	14.5	0	6	76.5
575	3	17	0	6	74
576	3	19.5	0	6	71.5
577	3	7	2.5	3.5	84
578	3	9.5	2.5	3.5	81.5
579	3	12	2.5	3.5	79
580	3	14.5	2.5	3.5	76.5
581	3	17	2.5	3.5	74
582	3	19.5	2.5	3.5	71.5

583	3	7	2.5	4	83.5
584	3	9.5	2.5	4	81
585	3	12	2.5	4	78.5
586	3	14.5	2.5	4	76
587	3	17	2.5	4	73.5
588	3	19.5	2.5	4	71
589	3	7	2.5	4.5	83
590	3	9.5	2.5	4.5	80.5
591	3	12	2.5	4.5	78
592	3	14.5	2.5	4.5	75.5
593	3	17	2.5	4.5	73
594	3	19.5	2.5	4.5	70.5
595	3	7	2.5	5	82.5
596	3	9.5	2.5	5	80
597	3	12	2.5	5	77.5
598	3	14.5	2.5	5	75
599	3	17	2.5	5	72.5
600	3	19.5	2.5	5	70
601	3	7	2.5	5.5	82
602	3	9.5	2.5	5.5	79.5
603	3	12	2.5	5.5	77
604	3	14.5	2.5	5.5	74.5
605	3	17	2.5	5.5	72
606	3	19.5	2.5	5.5	69.5
607	3	7	2.5	6	81.5
608	3	9.5	2.5	6	79
609	3	12	2.5	6	76.5
610	3	14.5	2.5	6	74
611	3	17	2.5	6	71.5
612	3	19.5	2.5	6	69
613	3	7	5	3.5	81.5
614	3	9.5	5	3.5	79
615	3	12	5	3.5	76.5
616	3	14.5	5	3.5	74
617	3	17	5	3.5	71.5
618	3	19.5	5	3.5	69
619	3	7	5	4	81
620	3	9.5	5	4	78.5
621	3	12	5	4	76
622	3	14.5	5	4	73.5
623	3	17	5	4	71
624	3	19.5	5	4	68.5
625	3	7	5	4.5	80.5
626	3	9.5	5	4.5	78

627	3	12	5	4.5	75.5
628	3	14.5	5	4.5	73
629	3	17	5	4.5	70.5
630	3	19.5	5	4.5	68
631	3	7	5	5	80
632	3	9.5	5	5	77.5
633	3	12	5	5	75
634	3	14.5	5	5	72.5
635	3	17	5	5	70
636	3	19.5	5	5	67.5
637	3	7	5	5.5	79.5
638	3	9.5	5	5.5	77
639	3	12	5	5.5	74.5
640	3	14.5	5	5.5	72
641	3	17	5	5.5	69.5
642	3	19.5	5	5.5	67
643	3	7	5	6	79
644	3	9.5	5	6	76.5
645	3	12	5	6	74
646	3	14.5	5	6	71.5
647	3	17	5	6	69
648	3	19.5	5	6	66.5
649	3	7	7.5	3.5	79
650	3	9.5	7.5	3.5	76.5
651	3	12	7.5	3.5	74
652	3	14.5	7.5	3.5	71.5
653	3	17	7.5	3.5	69
654	3	19.5	7.5	3.5	66.5
655	3	7	7.5	4	78.5
656	3	9.5	7.5	4	76
657	3	12	7.5	4	73.5
658	3	14.5	7.5	4	71
659	3	17	7.5	4	68.5
660	3	19.5	7.5	4	66
661	3	7	7.5	4.5	78
662	3	9.5	7.5	4.5	75.5
663	3	12	7.5	4.5	73
664	3	14.5	7.5	4.5	70.5
665	3	17	7.5	4.5	68
666	3	19.5	7.5	4.5	65.5
667	3	7	7.5	5	77.5
668	3	9.5	7.5	5	75
669	3	12	7.5	5	72.5
670	3	14.5	7.5	5	70

671	3	17	7.5	5	67.5
672	3	19.5	7.5	5	65
673	3	7	7.5	5.5	77
674	3	9.5	7.5	5.5	74.5
675	3	12	7.5	5.5	72
676	3	14.5	7.5	5.5	69.5
677	3	17	7.5	5.5	67
678	3	19.5	7.5	5.5	64.5
679	3	7	7.5	6	76.5
680	3	9.5	7.5	6	74
681	3	12	7.5	6	71.5
682	3	14.5	7.5	6	69
683	3	17	7.5	6	66.5
684	3	19.5	7.5	6	64
685	3	7	10	3.5	76.5
686	3	9.5	10	3.5	74
687	3	12	10	3.5	71.5
688	3	14.5	10	3.5	69
689	3	17	10	3.5	66.5
690	3	19.5	10	3.5	64
691	3	7	10	4	76
692	3	9.5	10	4	73.5
693	3	12	10	4	71
694	3	14.5	10	4	68.5
695	3	17	10	4	66
696	3	19.5	10	4	63.5
697	3	7	10	4.5	75.5
698	3	9.5	10	4.5	73
699	3	12	10	4.5	70.5
700	3	14.5	10	4.5	68
701	3	17	10	4.5	65.5
702	3	19.5	10	4.5	63
703	3	7	10	5	75
704	3	9.5	10	5	72.5
705	3	12	10	5	70
706	3	14.5	10	5	67.5
707	3	17	10	5	65
708	3	19.5	10	5	62.5
709	3	7	10	5.5	74.5
710	3	9.5	10	5.5	72
711	3	12	10	5.5	69.5
712	3	14.5	10	5.5	67
713	3	17	10	5.5	64.5
714	3	19.5	10	5.5	62

715	3	7	10	6	74
716	3	9.5	10	6	71.5
717	3	12	10	6	69
718	3	14.5	10	6	66.5
719	3	17	10	6	64
720	3	19.5	10	6	61.5
721	4	7	0	3.5	85.5
722	4	9.5	0	3.5	83
723	4	12	0	3.5	80.5
724	4	14.5	0	3.5	78
725	4	17	0	3.5	75.5
726	4	19.5	0	3.5	73
727	4	7	0	4	85
728	4	9.5	0	4	82.5
729	4	12	0	4	80
730	4	14.5	0	4	77.5
731	4	17	0	4	75
732	4	19.5	0	4	72.5
733	4	7	0	4.5	84.5
734	4	9.5	0	4.5	82
735	4	12	0	4.5	79.5
736	4	14.5	0	4.5	77
737	4	17	0	4.5	74.5
738	4	19.5	0	4.5	72
739	4	7	0	5	84
740	4	9.5	0	5	81.5
741	4	12	0	5	79
742	4	14.5	0	5	76.5
743	4	17	0	5	74
744	4	19.5	0	5	71.5
745	4	7	0	5.5	83.5
746	4	9.5	0	5.5	81
747	4	12	0	5.5	78.5
748	4	14.5	0	5.5	76
749	4	17	0	5.5	73.5
750	4	19.5	0	5.5	71
751	4	7	0	6	83
752	4	9.5	0	6	80.5
753	4	12	0	6	78
754	4	14.5	0	6	75.5
755	4	17	0	6	73
756	4	19.5	0	6	70.5
757	4	7	2.5	3.5	83
758	4	9.5	2.5	3.5	80.5

759	4	12	2.5	3.5	78
760	4	14.5	2.5	3.5	75.5
761	4	17	2.5	3.5	73
762	4	19.5	2.5	3.5	70.5
763	4	7	2.5	4	82.5
764	4	9.5	2.5	4	80
765	4	12	2.5	4	77.5
766	4	14.5	2.5	4	75
767	4	17	2.5	4	72.5
768	4	19.5	2.5	4	70
769	4	7	2.5	4.5	82
770	4	9.5	2.5	4.5	79.5
771	4	12	2.5	4.5	77
772	4	14.5	2.5	4.5	74.5
773	4	17	2.5	4.5	72
774	4	19.5	2.5	4.5	69.5
775	4	7	2.5	5	81.5
776	4	9.5	2.5	5	79
777	4	12	2.5	5	76.5
778	4	14.5	2.5	5	74
779	4	17	2.5	5	71.5
780	4	19.5	2.5	5	69
781	4	7	2.5	5.5	81
782	4	9.5	2.5	5.5	78.5
783	4	12	2.5	5.5	76
784	4	14.5	2.5	5.5	73.5
785	4	17	2.5	5.5	71
786	4	19.5	2.5	5.5	68.5
787	4	7	2.5	6	80.5
788	4	9.5	2.5	6	78
789	4	12	2.5	6	75.5
790	4	14.5	2.5	6	73
791	4	17	2.5	6	70.5
792	4	19.5	2.5	6	68
793	4	7	5	3.5	80.5
794	4	9.5	5	3.5	78
795	4	12	5	3.5	75.5
796	4	14.5	5	3.5	73
797	4	17	5	3.5	70.5
798	4	19.5	5	3.5	68
799	4	7	5	4	80
800	4	9.5	5	4	77.5
801	4	12	5	4	75
802	4	14.5	5	4	72.5

803	4	17	5	4	70
804	4	19.5	5	4	67.5
805	4	7	5	4.5	79.5
806	4	9.5	5	4.5	77
807	4	12	5	4.5	74.5
808	4	14.5	5	4.5	72
809	4	17	5	4.5	69.5
810	4	19.5	5	4.5	67
811	4	7	5	5	79
812	4	9.5	5	5	76.5
813	4	12	5	5	74
814	4	14.5	5	5	71.5
815	4	17	5	5	69
816	4	19.5	5	5	66.5
817	4	7	5	5.5	78.5
818	4	9.5	5	5.5	76
819	4	12	5	5.5	73.5
820	4	14.5	5	5.5	71
821	4	17	5	5.5	68.5
822	4	19.5	5	5.5	66
823	4	7	5	6	78
824	4	9.5	5	6	75.5
825	4	12	5	6	73
826	4	14.5	5	6	70.5
827	4	17	5	6	68
828	4	19.5	5	6	65.5
829	4	7	7.5	3.5	78
830	4	9.5	7.5	3.5	75.5
831	4	12	7.5	3.5	73
832	4	14.5	7.5	3.5	70.5
833	4	17	7.5	3.5	68
834	4	19.5	7.5	3.5	65.5
835	4	7	7.5	4	77.5
836	4	9.5	7.5	4	75
837	4	12	7.5	4	72.5
838	4	14.5	7.5	4	70
839	4	17	7.5	4	67.5
840	4	19.5	7.5	4	65
841	4	7	7.5	4.5	77
842	4	9.5	7.5	4.5	74.5
843	4	12	7.5	4.5	72
844	4	14.5	7.5	4.5	69.5
845	4	17	7.5	4.5	67
846	4	19.5	7.5	4.5	64.5

847	4	7	7.5	5	76.5
848	4	9.5	7.5	5	74
849	4	12	7.5	5	71.5
850	4	14.5	7.5	5	69
851	4	17	7.5	5	66.5
852	4	19.5	7.5	5	64
853	4	7	7.5	5.5	76
854	4	9.5	7.5	5.5	73.5
855	4	12	7.5	5.5	71
856	4	14.5	7.5	5.5	68.5
857	4	17	7.5	5.5	66
858	4	19.5	7.5	5.5	63.5
859	4	7	7.5	6	75.5
860	4	9.5	7.5	6	73
861	4	12	7.5	6	70.5
862	4	14.5	7.5	6	68
863	4	17	7.5	6	65.5
864	4	19.5	7.5	6	63
865	4	7	10	3.5	75.5
866	4	9.5	10	3.5	73
867	4	12	10	3.5	70.5
868	4	14.5	10	3.5	68
869	4	17	10	3.5	65.5
870	4	19.5	10	3.5	63
871	4	7	10	4	75
872	4	9.5	10	4	72.5
873	4	12	10	4	70
874	4	14.5	10	4	67.5
875	4	17	10	4	65
876	4	19.5	10	4	62.5
877	4	7	10	4.5	74.5
878	4	9.5	10	4.5	72
879	4	12	10	4.5	69.5
880	4	14.5	10	4.5	67
881	4	17	10	4.5	64.5
882	4	19.5	10	4.5	62
883	4	7	10	5	74
884	4	9.5	10	5	71.5
885	4	12	10	5	69
886	4	14.5	10	5	66.5
887	4	17	10	5	64
888	4	19.5	10	5	61.5
889	4	7	10	5.5	73.5
890	4	9.5	10	5.5	71

891	4	12	10	5.5	68.5
892	4	14.5	10	5.5	66
893	4	17	10	5.5	63.5
894	4	19.5	10	5.5	61
895	4	7	10	6	73
896	4	9.5	10	6	70.5
897	4	12	10	6	68
898	4	14.5	10	6	65.5
899	4	17	10	6	63
900	4	19.5	10	6	60.5
901	5	7	0	3.5	84.5
902	5	9.5	0	3.5	82
903	5	12	0	3.5	79.5
904	5	14.5	0	3.5	77
905	5	17	0	3.5	74.5
906	5	19.5	0	3.5	72
907	5	7	0	4	84
908	5	9.5	0	4	81.5
909	5	12	0	4	79
910	5	14.5	0	4	76.5
911	5	17	0	4	74
912	5	19.5	0	4	71.5
913	5	7	0	4.5	83.5
914	5	9.5	0	4.5	81
915	5	12	0	4.5	78.5
916	5	14.5	0	4.5	76
917	5	17	0	4.5	73.5
918	5	19.5	0	4.5	71
919	5	7	0	5	83
920	5	9.5	0	5	80.5
921	5	12	0	5	78
922	5	14.5	0	5	75.5
923	5	17	0	5	73
924	5	19.5	0	5	70.5
925	5	7	0	5.5	82.5
926	5	9.5	0	5.5	80
927	5	12	0	5.5	77.5
928	5	14.5	0	5.5	75
929	5	17	0	5.5	72.5
930	5	19.5	0	5.5	70
931	5	7	0	6	82
932	5	9.5	0	6	79.5
933	5	12	0	6	77
934	5	14.5	0	6	74.5

935	5	17	0	6	72
936	5	19.5	0	6	69.5
937	5	7	2.5	3.5	82
938	5	9.5	2.5	3.5	79.5
939	5	12	2.5	3.5	77
940	5	14.5	2.5	3.5	74.5
941	5	17	2.5	3.5	72
942	5	19.5	2.5	3.5	69.5
943	5	7	2.5	4	81.5
944	5	9.5	2.5	4	79
945	5	12	2.5	4	76.5
946	5	14.5	2.5	4	74
947	5	17	2.5	4	71.5
948	5	19.5	2.5	4	69
949	5	7	2.5	4.5	81
950	5	9.5	2.5	4.5	78.5
951	5	12	2.5	4.5	76
952	5	14.5	2.5	4.5	73.5
953	5	17	2.5	4.5	71
954	5	19.5	2.5	4.5	68.5
955	5	7	2.5	5	80.5
956	5	9.5	2.5	5	78
957	5	12	2.5	5	75.5
958	5	14.5	2.5	5	73
959	5	17	2.5	5	70.5
960	5	19.5	2.5	5	68
961	5	7	2.5	5.5	80
962	5	9.5	2.5	5.5	77.5
963	5	12	2.5	5.5	75
964	5	14.5	2.5	5.5	72.5
965	5	17	2.5	5.5	70
966	5	19.5	2.5	5.5	67.5
967	5	7	2.5	6	79.5
968	5	9.5	2.5	6	77
969	5	12	2.5	6	74.5
970	5	14.5	2.5	6	72
971	5	17	2.5	6	69.5
972	5	19.5	2.5	6	67
973	5	7	5	3.5	79.5
974	5	9.5	5	3.5	77
975	5	12	5	3.5	74.5
976	5	14.5	5	3.5	72
977	5	17	5	3.5	69.5
978	5	19.5	5	3.5	67

979	5	7	5	4	79
980	5	9.5	5	4	76.5
981	5	12	5	4	74
982	5	14.5	5	4	71.5
983	5	17	5	4	69
984	5	19.5	5	4	66.5
985	5	7	5	4.5	78.5
986	5	9.5	5	4.5	76
987	5	12	5	4.5	73.5
988	5	14.5	5	4.5	71
989	5	17	5	4.5	68.5
990	5	19.5	5	4.5	66
991	5	7	5	5	78
992	5	9.5	5	5	75.5
993	5	12	5	5	73
994	5	14.5	5	5	70.5
995	5	17	5	5	68
996	5	19.5	5	5	65.5
997	5	7	5	5.5	77.5
998	5	9.5	5	5.5	75
999	5	12	5	5.5	72.5
1000	5	14.5	5	5.5	70
1001	5	17	5	5.5	67.5
1002	5	19.5	5	5.5	65
1003	5	7	5	6	77
1004	5	9.5	5	6	74.5
1005	5	12	5	6	72
1006	5	14.5	5	6	69.5
1007	5	17	5	6	67
1008	5	19.5	5	6	64.5
1009	5	7	7.5	3.5	77
1010	5	9.5	7.5	3.5	74.5
1011	5	12	7.5	3.5	72
1012	5	14.5	7.5	3.5	69.5
1013	5	17	7.5	3.5	67
1014	5	19.5	7.5	3.5	64.5
1015	5	7	7.5	4	76.5
1016	5	9.5	7.5	4	74
1017	5	12	7.5	4	71.5
1018	5	14.5	7.5	4	69
1019	5	17	7.5	4	66.5
1020	5	19.5	7.5	4	64
1021	5	7	7.5	4.5	76
1022	5	9.5	7.5	4.5	73.5

1023	5	12	7.5	4.5	71
1024	5	14.5	7.5	4.5	68.5
1025	5	17	7.5	4.5	66
1026	5	19.5	7.5	4.5	63.5
1027	5	7	7.5	5	75.5
1028	5	9.5	7.5	5	73
1029	5	12	7.5	5	70.5
1030	5	14.5	7.5	5	68
1031	5	17	7.5	5	65.5
1032	5	19.5	7.5	5	63
1033	5	7	7.5	5.5	75
1034	5	9.5	7.5	5.5	72.5
1035	5	12	7.5	5.5	70
1036	5	14.5	7.5	5.5	67.5
1037	5	17	7.5	5.5	65
1038	5	19.5	7.5	5.5	62.5
1039	5	7	7.5	6	74.5
1040	5	9.5	7.5	6	72
1041	5	12	7.5	6	69.5
1042	5	14.5	7.5	6	67
1043	5	17	7.5	6	64.5
1044	5	19.5	7.5	6	62
1045	5	7	10	3.5	74.5
1046	5	9.5	10	3.5	72
1047	5	12	10	3.5	69.5
1048	5	14.5	10	3.5	67
1049	5	17	10	3.5	64.5
1050	5	19.5	10	3.5	62
1051	5	7	10	4	74
1052	5	9.5	10	4	71.5
1053	5	12	10	4	69
1054	5	14.5	10	4	66.5
1055	5	17	10	4	64
1056	5	19.5	10	4	61.5
1057	5	7	10	4.5	73.5
1058	5	9.5	10	4.5	71
1059	5	12	10	4.5	68.5
1060	5	14.5	10	4.5	66
1061	5	17	10	4.5	63.5
1062	5	19.5	10	4.5	61
1063	5	7	10	5	73
1064	5	9.5	10	5	70.5
1065	5	12	10	5	68
1066	5	14.5	10	5	65.5

1067	5	17	10	5	63
1068	5	19.5	10	5	60.5
1069	5	7	10	5.5	72.5
1070	5	9.5	10	5.5	70
1071	5	12	10	5.5	67.5
1072	5	14.5	10	5.5	65
1073	5	17	10	5.5	62.5
1074	5	19.5	10	5.5	60
1075	5	7	10	6	72
1076	5	9.5	10	6	69.5
1077	5	12	10	6	67
1078	5	14.5	10	6	64.5
1079	5	17	10	6	62
1080	5	19.5	10	6	59.5
